# Anterior Segment Imaging in Combat Ocular Trauma

**DOI:** 10.1155/2013/308259

**Published:** 2013-09-29

**Authors:** Denise S. Ryan, Rose K. Sia, Marcus Colyer, Richard D. Stutzman, Keith J. Wroblewski, Michael J. Mines, Kraig S. Bower

**Affiliations:** ^1^U.S. Army Warfighter Refractive Surgery Research Center at Fort Belvoir, Fort Belvoir, VA 22060, USA; ^2^Ophthalmology, Walter Reed National Military Medical Center, Bethesda, MD 20889, USA; ^3^The Wilmer Eye Institute, Johns Hopkins University, Baltimore, MD 21093, USA

## Abstract

*Purpose*. To evaluate the use of ocular imaging to enhance management and diagnosis of war-related anterior segment ocular injuries. *Methods*. This study was a prospective observational case series from an ongoing IRB-approved combat ocular trauma tracking study. Subjects with anterior segment ocular injury were imaged, when possible, using anterior segment optical coherence tomography (AS-OCT), confocal microscopy (CM), and slit lamp biomicroscopy. *Results*. Images captured from participants with combat ocular trauma on different systems provided comprehensive and alternate views of anterior segment injury to investigators. *Conclusion*. In combat-related trauma of the anterior segment, adjunct image acquisition enhances slit lamp examination and enables real time *In vivo* observation of the cornea facilitating injury characterization, progression, and management.

## 1. Introduction

Improvised explosive devices, rocket propelled grenades, and mortars expose a large number of soldiers and civilians to dangerous blasts that can damage the eye despite its small surface area [1]. Fragmentary projectiles, a secondary blast effect, are the most common cause of ocular injuries resulting in open-globe and adnexal lacerations [1–8]. The extent of injury from penetrating fragments depends on the size and velocity of the fragment, the depth of penetration, and site of impact [5]. While comprehensive knowledge of the patient's history and ocular examination is critical in the management of ocular injuries, ocular imaging modalities add valuable information for the course of clinical and surgical care of wounded soldiers. Furthermore, current imaging tools can monitor corneal wound healing, foreign body location, and if left in the cornea, foreign body migration. 

Anterior segment optical coherence tomography (AS-OCT) provides qualitative and quantitative assessment of the anterior segment [9]. Advantages of the AS-OCT include the noncontact capture system that causes minimal discomfort in trauma patients while providing high resolution cross-sections of the anterior segment. Corneal foreign body images provide information regarding the foreign body type (i.e., metallic versus nonmetallic), location, size, and depth. 

Another valuable imaging tool is the confocal corneal microscope, which can visualize cellular changes in the cornea. As seen in studies on donor corneas by Bourne and McLaren [10] and Waring et al. [11], the microscopic analysis of endothelial cells provides morphological characteristics that can be used to gauge corneal health. Also, a review by Jalbert et al. [12] showed that changes of *in vivo* cellular appearance could be used to characterize the corneal healing response. 

Previous studies have examined the extent of injury and wound healing characteristics of eyes subjected to trauma [1–8]. The current study illustrates the use of AS-OCT and confocal microscopy (CM), as a complement to slit lamp biomicroscopy, to observe detailed corneal changes following combat injury.

## 2. Methods

The combat ocular trauma tracking study is an ongoing Walter Reed National Military Medical Center Institutional Review Board-approved observational study. After informed consent, participants underwent detailed history taking and essential ophthalmologic examination. When possible, ancillary diagnostic tests, such as ocular imaging, were performed including noncontact anterior segment optical coherence tomography (AS-OCT; Visante, Carl Zeiss Meditec, Dublin, California, USA) and confocal microscopy (CM; Confoscan 4, Nidek, Gamagori, Japan).

A standard exam protocol to capture images was attempted for each subject. For the AS-OCT the following scans were obtained: anterior chamber quad (four evenly spaced scans), pachymetry map (eight evenly spaced scans), and high-resolution corneal quad (four evenly spaced scans). When feasible, specific injuries were targeted for additional scanning to complement slit lamp biomicroscopy images. Serial images were captured at subsequent visits as necessary. 

For the confocal microscope, scans were performed using a 40x lens providing real-time image capture of all corneal layers. Hydroxypropyl methylcellulose 0.3% gel was used as a coupling medium. The best focused and most representative endothelial cell image was selected for automated endothelial cell density analysis. When possible, specific injuries were targeted for additional image capture.

## 3. Results

**Figure 1:**
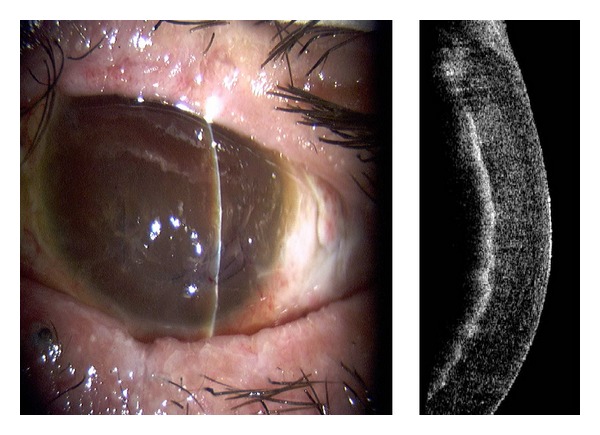
Slit lamp photograph and corresponding AS-OCT image of corneal blood staining.

**Figure 2:**
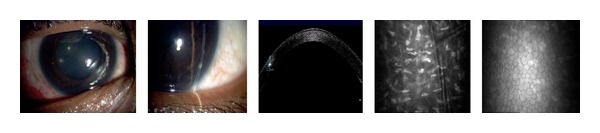
Slit lamp photographs of corneal foreign bodies and corresponding corneal AS-OCT and CM images of the stroma and endothelium.

**Figure 3:**
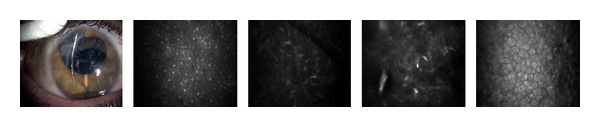
Slit lamp photograph and corresponding CM images of the epithelium, stroma, and endothelium with multiple corneal foreign bodies.

**Figure 4:**
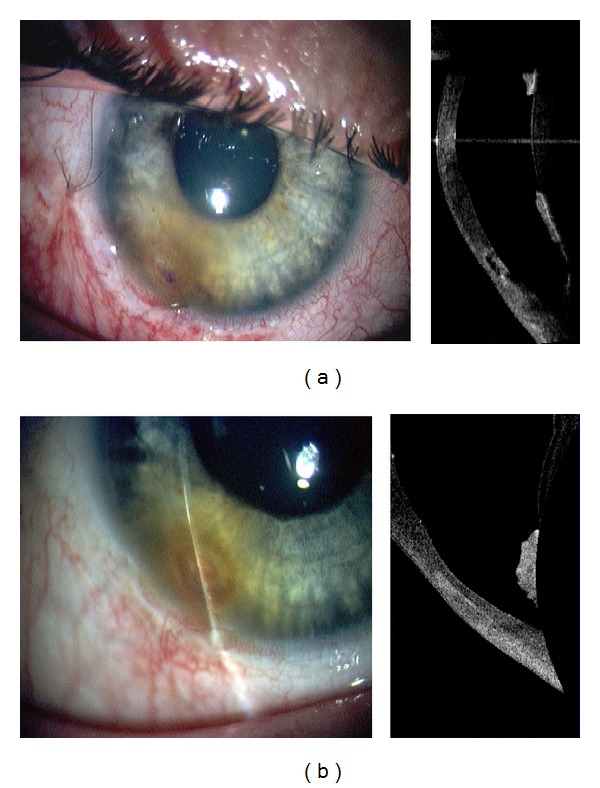
(a) Slit lamp photograph and corresponding AS-OCT of metallic foreign body. (b) Slit lamp photograph and corresponding AS-OCT of resulting rust ring and thinning after foreign body removal.

**Figure 5:**
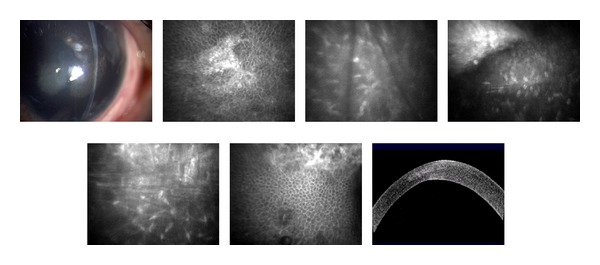
Traumatic corneal edema with central scarring three months after blast exposure. Epithelial scarring, Descemet's membrane folds, endothelial scar, and stromal edema and folds.

**Figure 6:**
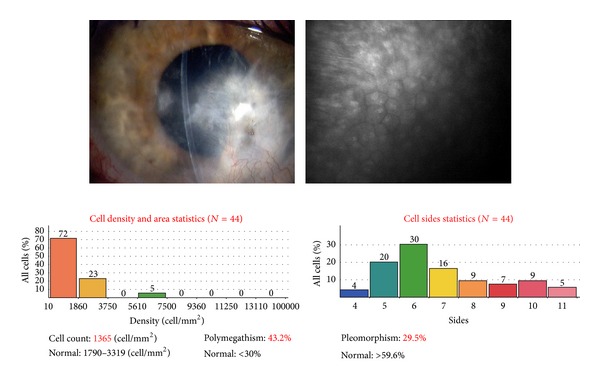
Endothelial cell changes eight months post blast exposure.

**Figure 7:**
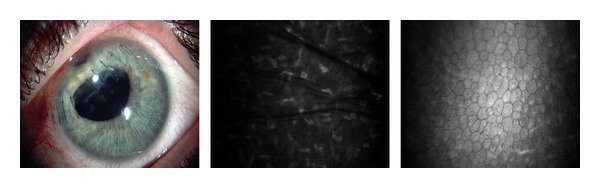
Corneal laceration, traumatic cataract with anterior capsule disruption status after laceration repair, and cataract extraction with intraocular lens implantation.

**Figure 8:**
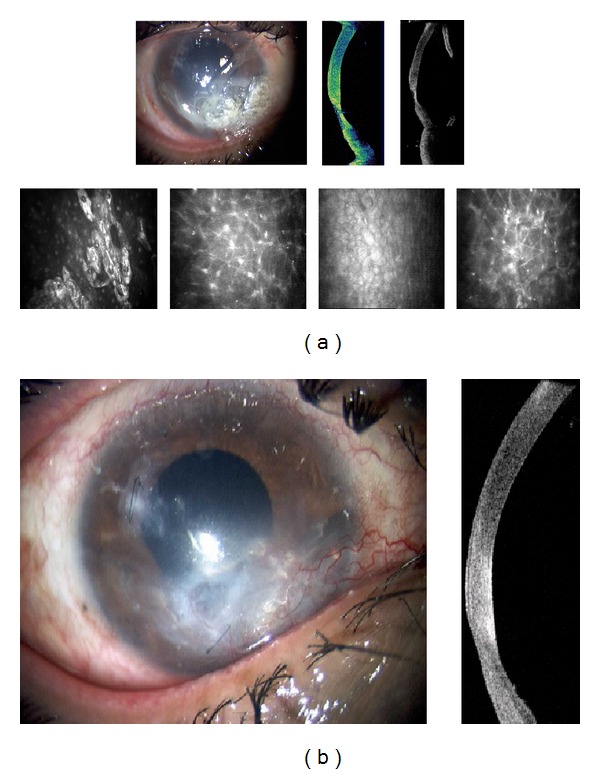
(a) Slit lamp photograph and corresponding AS-OCT and CM images ten days after injury and fungal keratitis (*Candida albicans*) with corneal thinning. Primary repair with sutures, tissue adhesive, and bandage contact lens. (b) Slit lamp photograph and corresponding AS-OCT and CM images of fungal keratitis after five weeks of treatment. Note improving corneal thickness and vascularized scar.

**Figure 9:**
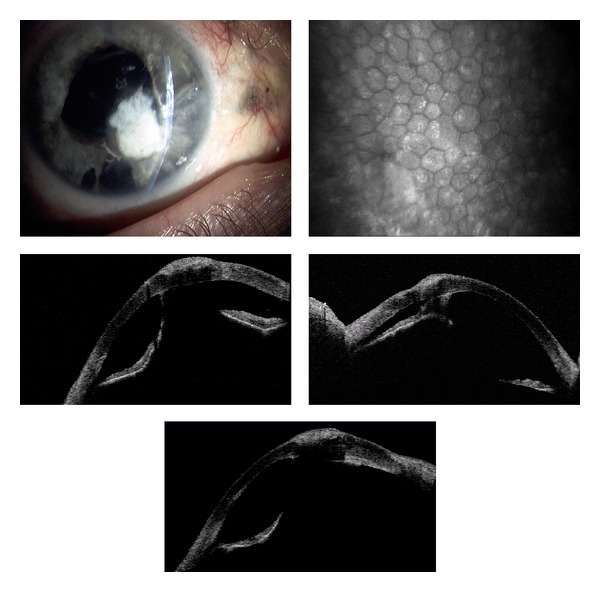
Corneal scar with processed pericardium graft and iris adhesions.

**Figure 10:**
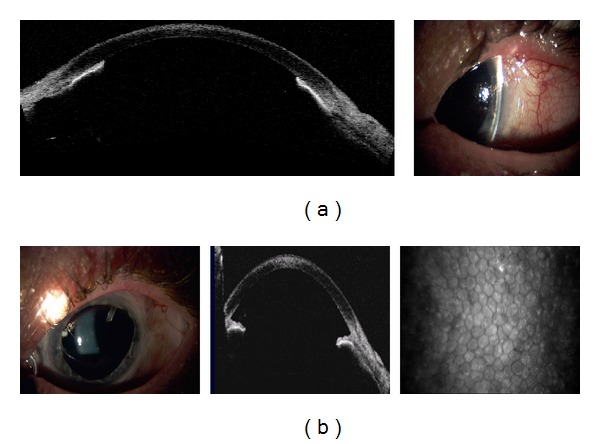
(a) A 360 degree iridocorneal adhesion after ocular injury. (b) Approximately six months later after surgical intervention.

**Figure 11:**
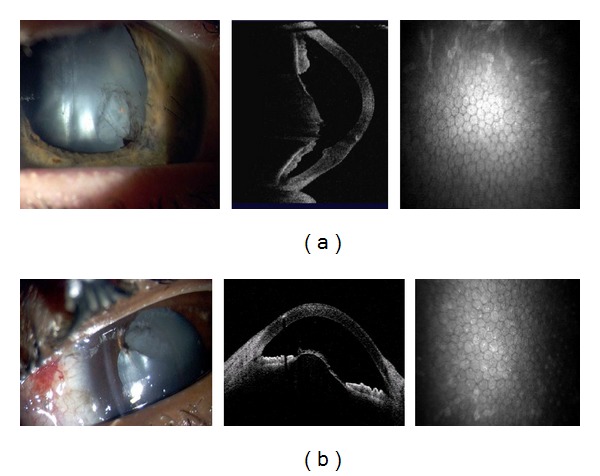
(a) Traumatic cataract with disruption of anterior lens capsule and corneal scar. (b) Intraocular foreign body and resulting traumatic cataract.

**Figure 12:**
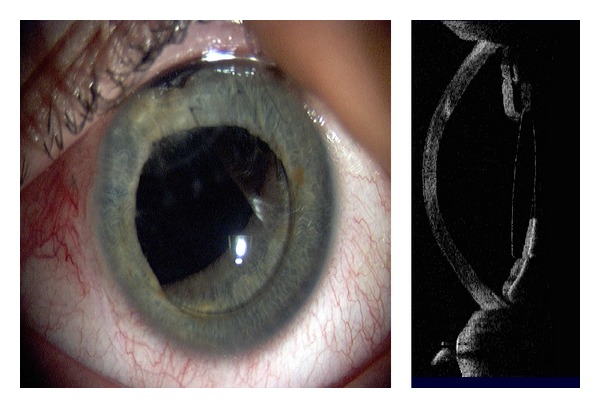
Subluxation of intraocular lens.

**Figure 13:**
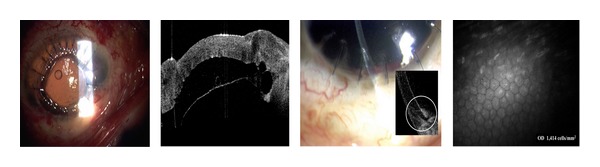
Aphakic eye with corneal graft and intraocular silicone oil.

This study reviewed captured clinical images and their associated anterior segment optical coherence tomography (AS-OCT) and confocal microscopy (CM) scans. One hundred four patients were imaged on the AS-OCT. Ninety-seven subjects with ocular injury were imaged using CM. This selection is a representative sampling of some of the anterior segment ocular trauma injuries and their complementary slit lamp examinations. Images of anterior segment trauma were divided into three main categories: cornea (Figures 1, 2, 3, 4, 5, 6, 7, 13), anterior chamber/anterior chamber angle/iris (Figures 8–11), and lens (Figures 10 and 12). AS-OCT and CM images and the corresponding slit lamp images are shown. 

Penetration by a foreign body from an improvised explosive device (IED) resulted in significant postsegment disruption.  Figure 1 shows a more detailed view of the resulting corneal decompensation and blood staining. Based on examination, imagery, and poor visual potential, no further reconstructive surgery was indicated. 

The multiple corneal foreign bodies observed in Figure 2 were also caused by an IED. AS-OCT imaging reveals size, location, and depth of the foreign body. Figures 2 and 3 illustrate the cellular changes of an ocular injury with multiple foreign bodies visualized by CM: irregular epithelium, inflamed stromal keratocytes, and an increase in pleomorphism. A deep stromal metallic foreign body with rust can be seen in Figure 4. A rust ring and corneal thinning are noted after removal. 

The wound healing response in an edematous cornea after blast exposure is demonstrated in the hyperreflective scars, stromal folds, Descemet's membrane folds, and endothelial changes in Figures 5–7.

Posttraumatic injury, a patient with a corneal laceration repaired with tissue adhesive was diagnosed with fungal keratitis (*Candida albicans*). Slit lamp, AS-OCT, and CM imaging seen in Figure 8(a) ten days after-injury illustrate almost 50% inferior thinning of the cornea, disruption of epithelial cells, linear septate fungal filaments and inflammatory cells, and endothelial irregularity. Repeated examination and imaging five weeks after treatment (Figure 8(b)) presents improved corneal thickness, scarring, and vascularization. 

After blast injury, a corneal laceration was repaired with processed pericardium graft that limited visibility. AS-OCT images in Figure 9 show iris irregularities behind the pericardium graft including adhesion to the endothelium and migration to the wound, with corresponding CM endothelial changes. In another case, AS-OCT confirmed slit lamp findings of 360-degree iridocorneal adhesion (Figure 10(a)). Subsequent imaging demonstrated improvement after surgical intervention.

Cornea, anterior chamber, and lens changes resulting from a traumatic cataract after blast injury are seen in Figure 11(a) with the hyperreflective corneal scar visible. AS-OCT imaging captures the anterior lens capsule rupture resulting from trauma in Figures 11(a) and 11(b). Three months posttraumatic corneal laceration repair, cataract extraction, and posterior chamber intraocular lens (IOL) implantation, IOL subluxation were reported (Figure 12). Figure 13 shows an aphakic eye with corneal graft and intraocular silicone oil after a penetrating injury caused corneal scarring, retinal detachment, lens dislocation, and traumatic iridectomy. 

## 4. Discussion

Because of the unique operational environment in the military, a comprehensive patient history and ocular examination are critical in the management of battle-related ocular injuries. Technology including computed tomography, magnetic resonance imaging, ultrasonography, [13, 14] optical coherence tomography (AS-OCT) [15], and confocal microscopy (CM) provides trauma surgeons with the ability to promptly view comprehensive and detailed images of an injury. Of the aforementioned imaging modalities, only AS-OCT and CM can be directly controlled and localized by the ophthalmologist allowing for real time *in vivo* examination, directed high resolution observation of the anterior segment, and sequential imaging, thereby facilitating the diagnosis and management of the ocular injury.

In this study, the noncontact scanning capability of the AS-OCT was significant in assessing fragile eyes, especially in patients with other facial injuries. AS-OCT uses 1310 nm wavelength to compare the light backscatter of tissue to a reference path, obtaining high-resolution anterior segment images at the expense of penetrating depth [16]. However, pigmentation, as observed in the iris, absorbs the incident light, preventing backscatter, thereby limiting visibility.  Figure 1 illustrates the highly refractive particles of a stroma infiltrated by red blood cell products migrating from the anterior chamber. While Figure 2 shows hyperreflective foreign bodies with shadowing occurring beyond, illustrating a limitation of AS-OCT. The size and depth of infiltration of foreign bodies can be measured with the AS-OCT, and if small and inert foreign bodies are left in place, their movement can be monitored in sequential images over time [17]. CM can also visualize corneal foreign bodies and the cellular changes surrounding them, as illustrated in Figure 3 and described by Jalbert et al. [12] and Figure 4. 

The corneal endothelium plays a critical role in maintaining corneal clarity by pumping fluid from the corneal stroma into the anterior chamber, and due to the limited regenerative ability of endothelial cells *in vivo* [18–20], cells subjected to trauma have been shown to migrate and enlarge to compensate for the loss of damaged cells [21]. Figures 3, 5–7 show endothelial cell damage in posttraumatic injured eyes, the true extent of which is unknown in some cases as preinjury images are not available. A report on CM by Kaufman et al. highlighted its use in the diagnosis of infectious keratitis, in quantifying the depth of foreign materials in the cornea, and in refractive surgery applications. As seen in Figure 8 of this study, CM images illustrate the cellular changes present in posttraumatic infectious keratitis and presence of fungal filaments as described by Vaddavalli et al. [22]. However, the necessity for contact and the difficulty in obtaining decentered full-thickness CM scans are disadvantages, especially, in eyes with anterior segment trauma in the periphery. Nonetheless, as seen in Figure 5, CM can capture images through edema [23], which is advantageous in these circumstances. Visualization of individual corneal layers may assist the surgeon in surgical planning when determining the method of corneal transplantation. Furthermore, CM can also be used to monitor graft health [24] as seen in Figure 13.

AS-OCT can also capture images through edematous or transparent tissue. More specifically, in Figure 9 it is difficult to discern iris detail beyond the pericardium graft in the slit lamp image whereas AS-OCT imaging provides a higher-resolution, more detailed view. Figures 10–13 further illustrate the anterior chamber, lens, and iris detail visible in anterior segment trauma patients. In addition, AS-OCT generates corneal thickness measurements that may not be possible by other noncontact methods due to tear film disturbance or surface irregularity. A study by Kim et al. [25] showed the repeatability and reproducibility for central corneal thickness using the AS-OCT. Figures 4, 8, and 12 illustrate corneal thinning and thickening with which repeated imagery at subsequent visits can be quantified and compared to monitor progression and rehabilitation.

In visually dependent specialties like ophthalmology, clinical imaging is essential, especially, when evaluating ocular trauma. Images in this study provided additional data to clinicians that may otherwise have not been known. Both AS-OCT and CM are important adjuncts to slit lamp examination, assisting the surgeon in decision making when assessing the health of the cornea and anterior segment. 

## References

[1] Thach AB, Johnson AJ, Carroll RB (2008). Severe eye injuries in the war in Iraq, 2003–2005. *Ophthalmology*.

[2] Thatch AB (2003). Eye injuries associated with terrorist bombings. *Ophthalmic Care of the Combat Casualty*.

[3] Shuker ST (2008). Mechanism and emergency management of blast eye/orbital injuries. *Expert Review of Ophthalmology*.

[4] Weichel ED, Colyer MH (2008). Combat ocular trauma and systemic injury. *Current Opinion in Ophthalmology*.

[5] Garner MJ, Brett SJ (2007). Mechanisms of injury by explosive devices. *Anesthesiology Clinics*.

[6] Gawande A (2004). Casualties of war—military care for the wounded from Iraq and Afghanistan. *The New England Journal of Medicine*.

[7] Zerihun N (1993). Blast injuries of the eye. *Tropical Doctor*.

[8] Ritenour AE, Baskin TW (2008). Primary blast injury: update on diagnosis and treatment. *Critical Care Medicine*.

[9] Doors M, Berendschot TTJM, de Brabander J, Webers CAB, Nuijts RMMA (2010). Value of optical coherence tomography for anterior segment surgery. *Journal of Cataract and Refractive Surgery*.

[10] Bourne WM, McLaren JW (2004). Clinical responses of the corneal endothelium. *Experimental Eye Research*.

[11] Waring GO, Bourne WM, Edelhauser HF, Kenyon KR (1982). The corneal endothelium. Normal and pathologic structure and function. *Ophthalmology*.

[12] Jalbert I, Stapleton F, Papas E, Sweeney DF, Coroneo M (2003). In vivo confocal microscopy of the human cornea. *British Journal of Ophthalmology*.

[13] Blice JP, Thatch A (2003). Imaging of ocular and adnexal truama. *Ophthalmic Care of the Combat Casualty*.

[14] Deramo VA, Shah GK, Baumal CR (1999). Ultrasound biomicroscopy as a tool for detecting and localizing occult foreign bodies after ocular trauma. *Ophthalmology*.

[15] Wylegala E, Dobrowolski D, Nowińska A, Tarnawska D (2009). Anterior segment optical coherence tomography in eye injuries. *Graefe’s Archive for Clinical and Experimental Ophthalmology*.

[16] Doors M, Berendschot TTJM, de Brabander J, Webers CAB, Nuijts RMMA (2010). Value of optical coherence tomography for anterior segment surgery. *Journal of Cataract and Refractive Surgery*.

[17] Fujimoto JG, Pitris C, Boppart SA, Brezinski ME (2000). Optical coherence tomography: an emerging technology for biomedical imaging and optical biopsy. *Neoplasia*.

[18] Doughty MJ (1989). Toward a quantitative analysis of corneal endothelial cell morphology: a review of techniques and their application. *Optometry and Vision Science*.

[19] Rozkowska AM, Colosi P, D’Angelo P, Ferreri G (2004). Age-related modifications of the corneal endothelium in adults. *International Ophthalmology*.

[20] Joyce NC, Harris DL, Mello DM (2002). Mechanisms of mitotic inhibition in corneal endothelium: contact inhibition and TGF-*β*2. *Investigative Ophthalmology and Visual Science*.

[21] Maurice DM (1972). The location of the fluid pump in the cornea. *Journal of Physiology*.

[22] Vaddavalli PK, Garg P, Sharma S, Sangwan VS, Rao GN, Thomas R (2011). Role of confocal microscopy in the diagnosis of fungal and acanthamoeba keratitis. *Ophthalmology*.

[23] Erie JC, McLaren JW, Patel SV (2009). Confocal microscopy in ophthalmology. *American Journal of Ophthalmology*.

[24] Niederer RL, Sherwin T, McGhee CNJ (2007). In vivo confocal microscopy of subepithelial infiltrates in human corneal transplant rejection. *Cornea*.

[25] Kim HY, Budenz DL, Lee PS, Feuer WJ, Barton K (2008). Comparison of central corneal thickness using anterior segment optical coherence tomography vs ultrasound pachymetry. *American Journal of Ophthalmology*.

